# First person – Angela Fan and Yoldas Yildiz

**DOI:** 10.1242/bio.062134

**Published:** 2025-07-15

## Abstract

First Person is a series of interviews with the first authors of a selection of papers published in Biology Open, helping researchers promote themselves alongside their papers. Angela Fan and Yoldas Yildiz are co-first authors on ‘ Ethanol induces subcellular trafficking of the RNA-binding protein, hnRNP A1, in neuronal cells *in vitro*, but not in the peripubertal rat brain’, published in BIO. Angela is a PhD candidate in the lab of Toni R. Pak at Loyola University Chicago Stritch School of Medicine, Maywood, IL, USA, investigating biomolecular condensates, particularly their formation mechanisms and functional roles under stress, especially in the context of pathophysiology. Yoldas is a PhD candidate in the same lab, investigating the cellular and molecular mechanisms of nuclear receptor signalling, how they change in ageing, and the downstream consequences to the transcriptome and proteome.


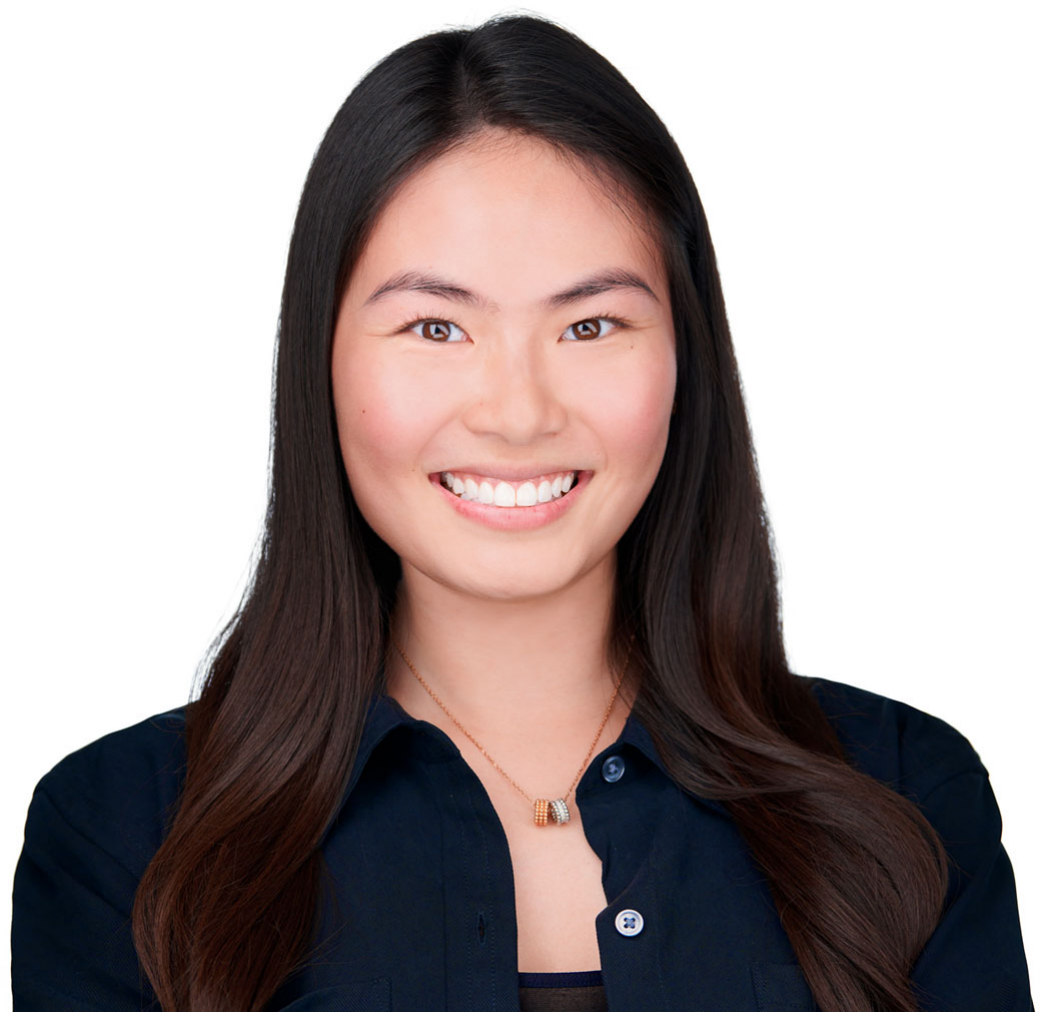


**Angela Fan**

**Describe your scientific journey and your current research focus**

**A.F.:** I've always been fascinated by the natural world – especially the ocean – which initially led me to major in marine biology during my undergraduate studies. As I became more involved in research, I realized that marine organisms can serve as powerful models for understanding environmental change, act as biomarkers, and even help us uncover mechanisms relevant to human health. Wanting to bridge my interests in marine and biomedical sciences, I joined labs that utilized zebrafish as a model system to study developmental biology, tissue regeneration, and to perform high-throughput screening for novel anesthetics. Those experiences sparked my desire to pursue more advanced training, which brought me to Loyola University Chicago's Integrated Program in Biomedical Sciences. I now have the privilege of conducting my PhD research under the mentorship of Dr Toni Pak. My current work adopts a more molecular perspective, where I investigate how RNA-binding proteins are altered in the brain following adolescent binge drinking, with a focus on their roles in post-transcriptional regulation during neurodevelopment.

**Y.Y.:** During my undergraduate studies, I became really interested in cell signaling and its role in development. This led me to pursue a Masters in reproductive and developmental biology, which began my interest passion in the field of neuroendocrinology, where my thesis project focused on the internalization of the luteinizing hormone receptor and its effect on downstream cell signaling. I worked for a few years in industry and then returned to academia to get my PhD under the mentorship of Dr Toni Pak. Currently, my research focus is on estrogen receptor beta signaling and the differences in healthy versus unhealthy aging and neurodegenerative diseases.

**Who or what inspired you to become a scientist?**

**A.F.:** One of the most influential figures in shaping my scientific perspective has been Dr Sylvia Earle, the renowned marine biologist and explorer. I had the opportunity to attend one of her seminars during my undergraduate studies, where she described the ocean as a deeply interconnected system – from microscope algae to expansive coral reefs – emphasizing how each component plays a vital role in maintaining ecological balance. What stood out most to me was her ability to communicate the urgency of ocean conservation through powerful storytelling.


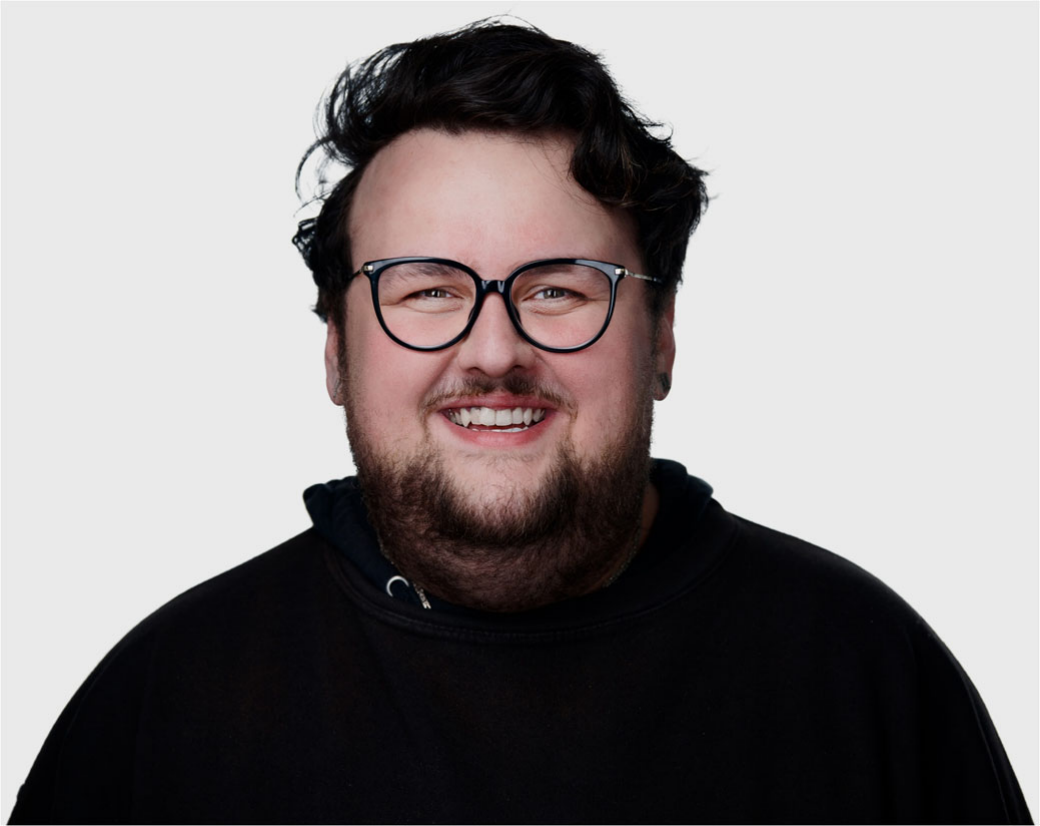


**Yoldas Yildiz**

That seminar left a lasting impression on me, and as I've progressed in my scientific career, her message continues to resonate. Whether in ocean research, biomedical science, or technology, I believe it's critical to bridge the gap between scientific discovery and public understanding. Communicating the ‘why’ behind our research – why it matters for human health, the environment, or society – is essential for inspiring informed action and broader support.

**Y.Y.:** From a very young age, I would attend my mother's doctor's appointments to translate for her and was exposed to how the body functions and how various conditions can be treated. I became fascinated by how and why the body becomes sick. I had an amazing high school teacher who inspired me to pursue a career in science and helped me apply to college in the United States. When I went to college, I took more advanced biology classes and began to understand how complex we are at the molecular level and wanted to delve even deeper…and here I am.

…both where in the brain and sex may influence how this protein [hnRNP A1] responds to alcohol during adolescence

**How would you explain the main finding of your paper?**

This study looked at how a protein called hnRNP A1 behaves in brain cells when exposed to alcohol during adolescence. Normally, this protein stays in the control center of the cell (the nucleus), but, under stress, it can move to other parts of the cell. The researchers found that when brain-like cells were exposed to alcohol in the lab, the protein moved out of the nucleus. However, this same effect did not happen in the brains of live adolescent rats that were exposed to alcohol. They also tested a byproduct of alcohol called acetate, but it didn't cause the protein to move either. Interestingly, when the protein moved in the lab-grown cells, the cells also became swollen, suggesting that the movement might be linked to the kind of stress that comes from changes in water or pressure inside the cell. Finally, the study discovered that this protein shows different levels in different parts of the brain, and these levels can vary between males and females. This suggests that both where in the brain and sex may influence how this protein responds to alcohol during adolescence.

**What are the potential implications of this finding for your field of research?**

In our study, we found that alcohol exposure causes the RNA-binding protein hnRNP A1 to move out of the nucleus in cultured neurons, but, interestingly, this didn't happen in the brains of adolescent rats. This told us that something about the real brain environment – like metabolism or brain region differences – might prevent that movement. We also noticed that when hnRNPA1 moved in the cells, the cells were swollen, which suggests that osmotic stress could be the real driver, not just the alcohol itself. That adds a new angle to how we think about stress responses in brain cells during adolescent binge drinking. Since hnRNPA1 helps regulate small RNAs that control gene expression, its movement to the cytoplasm could disrupt normal brain development. It might also interfere with stress granules – those protective structures cells form under stress – potentially affecting neuron survival. What's also interesting is that acetate, a byproduct of alcohol, didn't cause the same effect, so metabolism may actually protect against this protein movement *in vivo*. We found that hnRNP A1 levels vary by brain region and between males and females, which might help explain sex-based differences in how the brain responds to alcohol. Altogether, these results highlight the importance of studying alcohol effects in more realistic systems that reflect the complexity of the living brain. Broadly, our work shows that alcohol during adolescence can affect RNA regulation in very specific ways – depending on stress type, sex, and brain region – which could have long-term consequences for brain health.

…the beauty of science is that learning will never end so you can constantly feed your curiosity and there's never a boring day

**Figure:**
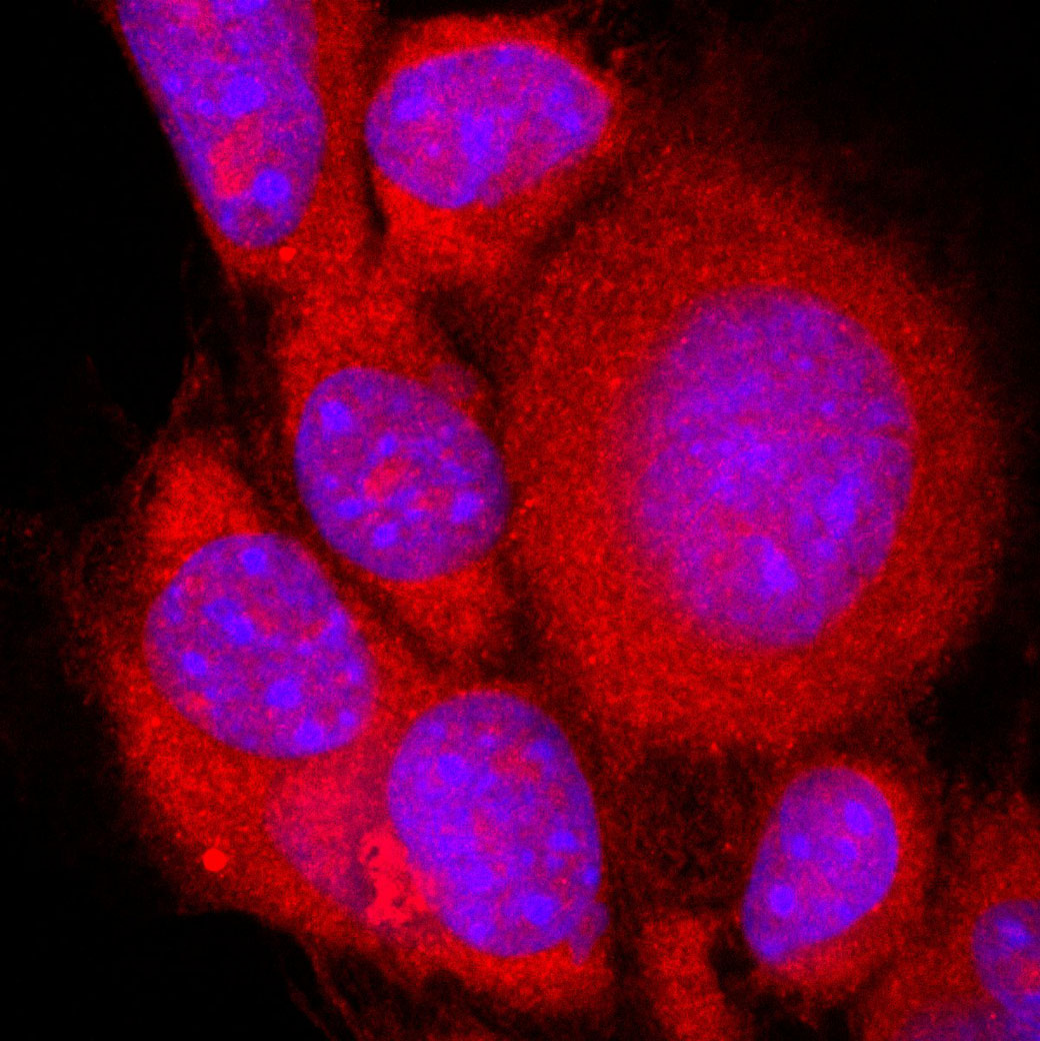
hnRNP A1 (red) typically resides in the nucleus (blue), but relocalizes to the cytoplasm after 2-h ethanol exposure in IVB cells (rat hypothalamic neuronal-derived cell line), and the cells also exhibit cellular swelling.

**Which part of this research project was the most rewarding?**

One of the most rewarding parts of this project was when we finally saw hnRNP A1 move from the nucleus to the cytoplasm in our neuronal cell model after alcohol exposure. That result was a key turning point – it confirmed our hypothesis and showed that alcohol can directly alter RNA-binding proteins under certain conditions. But what made the project even more interesting was that we didn't see the same effect in our *in vivo* experiments with adolescent rats. That contrast was eye-opening and highlighted how differently alcohol can act in a living brain compared to a controlled cell culture system. It really emphasized the importance of considering both models when studying alcohol's effects, especially in the context of complex systems like the brain. Presenting those findings at a conference was incredibly rewarding – not just because of the positive feedback, but also because it sparked meaningful conversations about how to bridge the gap between *in vitro* and *in vivo* research. It was a reminder that unexpected results can be just as important as confirming ones, and sometimes they push the science forward even more. That moment gave me a broader perspective on experimental design and interpretation. It also strengthened my appreciation for the complexity of neurobiology and alcohol research.

**What do you enjoy most about being an early-career researcher?**

**A.F.:** One of the things I value about my research experience is having the space to make mistakes and learn from them. As frustrating as it can be in the moment, each mistake is an opportunity to gain new insights, troubleshoot, and refine my approach. That process of adapting and optimizing has been essential to my growth as a scientist. I've also been fortunate to have a mentor and collaborators who are open to idea-sharing and regular discussions. Being able to bounce ideas off one another in a supportive environment makes the work both productive and genuinely enjoyable. It's exciting to think that some of those conversations could spark discoveries that contribute to the broader scientific community.

**Y.Y.:** I'm constantly learning. Both in my own research and through departmental seminars, journal clubs, day-to-day conversations with other researchers and, most importantly, my PI. I think the beauty of science is that learning will never end so you can constantly feed your curiosity and there's never a boring day. Even simple conversations with my PI turn into me learning something new about my field or a paper being sent to my email that I'd love to read.

**What piece of advice would you give to the next generation of researchers?**

**A.F.:** I've learned that failure is a natural and necessary part of the scientific process – as long as you use it as a stepping stone to move forward. Every setback is an opportunity to learn something new and refine your approach. In fact, I believe that the moment you stop failing, you're probably no longer pushing yourself to grow or explore new ideas.

**Y.Y.:** Persevere. You will encounter challenges throughout your entire career – from getting into a PhD program, conducting and troubleshooting experiments, struggling through classes, taking candidacy exams, publishing your research, and even when you become a young PI, as the focus shifts to securing grants and attracting students. Facing failure with perseverance and fighting through the struggles will teach you more about life in science than any textbook could. Also, surround yourself with ambitious people who constantly push you to be the best version of yourself.

**What's next for you?**

**A.F.:** My next step is to focus on the final two years of my PhD – refining my experimental approaches, gathering the remaining data and, ultimately, completing my dissertation. Two years might sound like a long time, but I know it will pass quickly. I'm excited to see how the project evolves and, just as importantly, how much I'll continue to grow as a scientist along the way.

**Y.Y.:** I'm currently entering my fifth year of my PhD. I'm seeking potential collaborators and future postdoctoral opportunities as I approach the completion of my degree within the next 1-1.5 years. I hope to stay in the field of endocrinology and nuclear receptors and hopefully run my very own lab in the future.
